# Financial Literacy in Orthopaedic Surgery Residents: A COERG Survey

**DOI:** 10.5435/JAAOSGlobal-D-21-00276

**Published:** 2022-02-08

**Authors:** Ryan J. Cone, Brent M. Cone, Kyle D. Paul, Alexandra M. Arguello, D. Michael McCalman, Gerald McGwin, Scott E. Porter, S. Elizabeth Ames, Michael D. Johnson, Brent A. Ponce, COERG

**Affiliations:** From the Department of Orthopaedics (Dr. R. J. Cone, Dr. B. M. Cone, Paul, Arguello, McCalman, and Dr. Johnson), and the Department of Epidemiology (Dr. McGwin), University of Alabama at Birmingham, Birmingham, AL; the Department of Orthopaedic Surgery, Prisma Health-Upstate, Greenville, SC (Dr. Porter); the Department of Orthopedics and Rehabilitation, University of Vermont Medical Center, Burlington, VT (Dr. Ames); and The Hughston Clinic, Columbus, GA (Dr. Ponce).

## Abstract

**Introduction:**

Financial literacy is the individual ability and skill to make informed decisions in the management of resources within the financial marketplace to yield a lifetime of financial well-being. Residents across several subspecialties have demonstrated low levels of financial literacy, and it is thought that more financial education is needed during residency training. The purpose of this study is to perform a comprehensive evaluation on financial literacy and financial attitudes of orthopaedic surgery residents. The authors hypothesize that orthopaedic residents will have low levels of financial literacy and financial satisfaction.

**Methods:**

A 46-question anonymous survey was administered through COERG (Collaborative Orthopaedic Educational Research Group) to 1028 orthopaedic surgery residents of all postgraduate year at 43 programs with broad national distribution. Resident demographics and survey responses regarding knowledge of finance and investment topics, application of financial principles, and personal financial status were compared.

**Results:**

The survey response rate was 48% (494/1028). The average financial literacy score of all orthopaedic resident participants was 60.9% (±16.5%). A total of 35.5% of orthopaedic residents were satisfied with their current financial situation. Saving for retirement and lower loan burdens correlated with greater financial satisfaction in financial situation. Scores were higher in orthopaedic residents with greater childhood annual household income, no credit card debt, higher levels of parent education, and active retirement savings plans.

**Conclusions:**

Orthopaedic residents show significant deficits in overall financial and investment knowledge combined with a dissatisfaction with financial situations while in residency. Orthopaedic residency programs have the opportunity to implement program-sponsored training and financial resources to enhance the resident education experience.

Financial literacy is the individual ability and skill to make informed decisions in the management of resources within the financial marketplace to yield a lifetime of financial well-being.^1,2^ This subject is part of systems-based practice and professionalism for physicians, and as such, it is an important education topic in residency training. While the concept of implementing financial literacy curricula in residency has been proposed, programs are challenged to balance educational time given broad requirements and rapid changes in surgical knowledge and techniques.^3^ Although instruction in financial literacy is not an Accreditation Council for Graduate Medical Education requirement, financial concerns are a reality for surgical residents.^3^ The Association of American Medical Colleges reports that the median amount of medical student debt before residency in 2018 is $200,000, which continues to increase annually.^4^ Demonstrated growth rates in medical school tuition over a 10-year period are twice as high as inflation.^5^ Although physicians in the United States have historically earned high, stable salaries, which theoretically have led to lower financial stress, associated medical school debt has outpaced adjustments in physician salary, creating a financial burden among physicians entering the workforce.^6^

Studies demonstrate that medical school graduates carry a large amount of educational debt in conjunction with having notable deficits in basic financial knowledge and skill.^7,8,9,10,11^ This combination can create a lack of financial management skills. Potential negative consequences include effects on specialty choice and other career decisions; debt incurred during medical training has been implicated in students not pursuing careers in primary care.^6,12-14^ Studies have also linked increasing financial burden to increased stress and burnout in the surgical resident population.^7,15,16^ In addition, consistent themes regarding financial knowledge of residents in all specialties^8,9,10,11^ have been reported in the literature. Residents demonstrate a general lack of financial knowledge and have a consistent sense of low overall financial preparedness.^9,16-18^ A mismatch between degree of debt and self-assessment of financial health may exist, and many residents think that more financial education is needed during residency training.^8,9,10,11^

There are no existing studies that evaluate financial literacy of residents within the field of orthopaedic surgery. The purpose of this study is to perform a comprehensive evaluation on financial literacy and financial attitudes of a large multiregional cohort of orthopaedic surgery residents. Our hypothesis is that orthopaedic residents will have low levels of financial literacy and financial satisfaction.

## Methods

### Study Population

A 46-question anonymous survey was administered to 1028 orthopaedic surgery residents of all postgraduate year (PGY) levels at 43 programs with broad national distribution from April 7 until June 7, 2020. Enrollment was through COERG (Collaborative Orthopaedic Educational Research Group) following institutional review board approval. Follow-up emails were sent to residents by their respective program directors twice. Survey responses were deidentified, collected, and managed using REDCap electronic data capture tools hosted at the senior author's institution.^19,20^ Each survey was required to have at least 80% of questions answered for inclusion.

### Survey Information

The survey included questions pertaining to resident demographics, knowledge of finance and investment topics, and application of financial principles/personal financial status. Demographic questions included age, PGY in training, marital status, presence of financially dependent children, city population, annual household income during childhood, and levels of parental education. Permission was granted to use the validated National Financial Capability Survey questions for this study by the Financial Industry Regulatory Authority Investor Education Foundation. The National Financial Capability Study by Financial Industry Regulatory Authority defines the categories of financial capability as making ends meet, planning ahead, managing financial products, and financial knowledge and decision making. Previously validated questions regarding application of financial principles and personal financial status were derived in part from the Ahmad et al^9^ 2017 survey of all resident specialties. A copy of the survey is provided (Appendix 1, http://links.lww.com/JG9/A189).

### Statistical Analysis

Data analysis was conducted by an experienced statistician using SAS software (SAS Institute). Respondent demographics and financial literacy survey response frequencies were calculated with descriptive statistics. Financial literacy score comparisons by category were analyzed with one-way analysis of variance testing. Significance was established at *P* = 0.05.

## Results

The survey response rate was 48% (494 of 1,028). The average financial literacy score of all orthopaedic resident participants was 60.9% (±16.5%). The average age of respondents was 29.9 years (±2.5 years), and residents studied were distributed evenly across PGY training (PGY1 to PGY5+). Respondents were more likely to be married (60.9%), without children (74.1%), and more likely to plan on entering private practice (44.5%, academic 18.4%). Resident demographics can be referenced in Supplemental Table I (http://links.lww.com/JG9/A186).

Most respondents reported spending less than their household income over the previous year, and 75.7% noted no difficulty covering monthly expenses. Most residents studied had student debt (77.5%). Of responding residents, 71.3% reported the ability to presently cover 3 months of expenses with savings. Less than 45% of residents had started saving for retirement. Of respondents, 33.8% had not undergone formal financial training, and 43.7% had not received formal practice management training. A complete display of response frequencies can be referenced in Supplemental Table II (http://links.lww.com/JG9/A187).

A total of 35.5% of orthopaedic residents were satisfied with their current financial situation based on survey response. Greater loan burden correlated with decreased satisfaction with the individual's financial situation (*P* < 0.0001), and saving for retirement was associated with greater satisfaction (*P* = 0.002). In addition, 23.5% of respondents reported satisfaction with either training in financial management or training in practice management (9.7% satisfied in both), and 52% declared that they would like more training in these areas. Almost 50% felt that their financial management training to that point was inadequate (Supplemental Table II, http://links.lww.com/JG9/A187).

Financial literacy scores varied with demographics. Scores were higher in orthopaedic residents with greater childhood annual household income (*P* = 0.02), no credit card debt (*P* = 0.01), higher levels of parent education (parent 1, *P* = 0.047; parent 2, *P* = 0.03), and active retirement savings plans (*P* < 0.001). Residents with a mortgage had trending but not significantly higher scores than those without a mortgage (62.2% with mortgage, 59.8% without mortgage, *P* = 0.11). No significant differences were found between PGY level of training (*P* = 0.55), age (*P* = 0.34), marital status (*P* = 0.48), presence of children (*P* = 0.75), or residents with student loan debt (*P* = 0.33). All financial literacy score averages based on differentiating factors with associated *P* values can be referenced in Supplemental Table III (http://links.lww.com/JG9/A188).

## Discussion

Financial literacy is recognized as important, but remains an overlooked area of surgical resident education in the setting of increasing educational debt and burnout. This survey study investigates the levels of financial literacy and financial satisfaction of orthopaedic residents. Respondents were found to have low financial literacy scores and reported low levels of retirement investments, high levels of educational debt, and a general dissatisfaction in financial situations. Opportunity exists to improve these areas by increasing surgical resident financial literacy through improvement of financial management training resources.

The average financial literacy score of 60.9% in our population demonstrates a lack of financial literacy among orthopaedic residents. These results are comparable to the study by Ahmad et al in which residents of all specialties scored an average of 52%.^9,21,22^ Our results were further stratified based on various demographic factors. Financial literacy scores did not change across PGY level, suggesting that orthopaedic residents are not increasing financial literacy during training in the absence of formal program curriculum. In addition, there was no difference in financial knowledge based on age, marital status, dependents, or student loan debt.

We found significant difference in test results based on existing credit card debt, retirement savings status, and childhood annual household income. Fewer than 15% of our respondents reported credit card debt. However, those that carried credit card debt had lower quiz score results. Credit card debt can be situational, but it may also represent unhealthy financial practices. This finding suggests a link between unhealthy financial practices (credit card debt) and lower financial knowledge. The opposite pattern was also found. Those who grew up in higher income families and with parents of higher education status displayed higher financial literacy. This finding correlates with previous literature, as studies have shown that early exposure to healthy financial practice and education leads to healthy fiscal practice and improved financial security.^23-25^

Orthopaedic residents with retirement savings scored significantly higher on the financial literacy quiz than those who had not begun saving for retirement, suggesting that those engaged in their financial situation with an active role in planning for the future display greater financial literacy. Interestingly, most orthopaedic residents practice healthy spending habits while in residency, as over two-thirds reported spending less than their income in a given year. Over 75% reported no difficulty covering monthly expenses, and almost 85% have an emergency fund with an ability to come up with $2,000 in a given month, suggesting that respondents demonstrate self-awareness about spending habits and can save money while in residency. However, less than half of respondents have begun saving for retirement, which may suggest that many residents may be deferring the planning of their financial futures until their training is completed. These are valuable years for establishing future savings, as time is one of the most useful tools for tax-free growth vehicles (like Roth IRAs) that have the potential for compounding growth over decades. As educational institutions, it would seem logical to provide the necessary resources and knowledge to engage residents in planning their financial futures. Furthermore, given the link between student debt and burnout, teaching residents to positively contribute to their financial health may quell the symptoms of burnout as it relates to financial stressors.^7,15,16^

The findings also suggest a void in financial education and training despite an apparent need and desire. Less than 40% of respondents reported satisfaction with their financial situation, and dissatisfaction correlated with lack of savings and increased debt burden. More than half of our study population carried a burden of over $200,000 of student loans, and almost 25% carried more than $300,000. This is consistent with the findings of a 2019 survey of orthopaedic residents where 40% of residents had greater than $200,000 in loan debt, and a 10-year trend of increasing student loan debt has been demonstrated for all medical school graduates.^11,26^ Fewer than 50% of respondents reported having access to financial education opportunities within their respective residency programs, and over 75% of respondents reported dissatisfaction with either their financial or practice management training. The percentage of residents dissatisfied with training is lower than the survey by Jennings et al^11^ (83%) but still represents most residents. Over two-thirds of respondents accessed outside resources within the past 6 months for financial education, such as consultants or online resources, which is concerning as orthopaedic residents may be unequipped to select an unbiased financial educational resource.

An opportunity exists for residency programs to provide basic financial knowledge to assist surgical residents in establishing healthy financial foundations. Ahmad et al^9^ recommend that financial education training for residents should at least include monthly budgeting; debt/loan management; credit score building; savings and retirement planning options; life, health and disability insurance; and estate planning strategies. Mizell et al^27^ implemented a comprehensive financial management curriculum for general surgery residents within grand rounds sessions at their institution resulting in a self-reported increase in financial knowledge and interest among the residents. Dhaliwal et al^17^ implemented a 90-minute seminar on personal finance, which showed improvements in investment strategies. Programs lacking in local experts could also suggest widely sourced resources, such as The White Coat Investor, followed by group discussions as part of a financial wellness program.^28,29^

Over half of the respondents in this study suggested a sit-down meeting with a personal financial advisor as the most effective intervention for effective financial training. A personal finance advisor can serve as a valuable resource for financial management, although concerns exist regarding the financial incentive and conflicts of interest for financial advisors, which may include annual fees or commission payments based on investments.^28^ Orthopaedic residents without proper financial knowledge or institutional oversight could be vulnerable targets of ill-intentioned advisors. It is imperative for these residents to have baseline financial knowledge to critically evaluate advisor services. Ideally, a training institution or department could provide staff financial advisors who are not incentivized by commissions and are focused on the education of their trainees, but this is not commonly available. Financial education is the next best option.

Our data suggest that most orthopaedic residents desire more program organized training in financial and practice management. A blanket recommendation for one specific financial literacy training method was not identified, nor was it the scope of this analysis. It seems that programs can implement specific steps to help orthopaedic residents improve their financial situation through training. Most residency programs are affiliated with large universities, which have associated business schools, offering opportunities for collaboration. The authors encourage either the creation of a standardized education platform focused on surgical trainees or the creation of a network of financial specialists to provide frequent and accessible educational resources on topics related to the management of personal finances and investments.

Several limitations to this study exist. The conclusions may not be applicable to every residency program, as not every orthopaedic residency program was represented; however, the authors made attempts to avoid convenience sampling bias by targeting 43 different, geographically diverse orthopaedic residency programs. Response bias may exist, as residents more concerned with financial literacy may have been more inclined to complete the survey, but our literacy scores are comparable with previous literature examining residents in all specialties. Precise determinations of resident financial knowledge are limited without the ability to incorporate exhaustive surveys on financial and investor literacy. The authors selected questions from a previously validated questionnaire that have been used to assess knowledge of basic financial topics in the areas of savings and investing.^9^ In addition, gender information was not identified in the survey to preserve anonymity. However, previous literature has demonstrated that gender may affect financial literacy.^30,31,32,33^ Although discussion on this topic is beyond the scope of this study, financial literacy training programs targeting populations at risk for lower financial literacy such as females are warranted, and additional research in this area of orthopaedics is necessary. Although not all orthopaedic residents responded to the survey, a 48% response rate is acceptable for supporting conclusions derived from acquired data. If all 534 nonrespondents achieved a financial literacy score of 100%, the mean overall score would be approximately 80%, which still demonstrates a gap in knowledge. In addition, our response rate of 48% is significantly higher than other comparable surveys in the literature.^9,11^ Future research in the area of financial literacy in orthopaedic residents is warranted. Studies addressing the improvements of scores following interventions will assist programs in better equipping their residents for financial success.

## Conclusions

Orthopaedic residents show significant deficits in overall financial and investment knowledge combined with a dissatisfaction with financial situations while in residency. The surveyed residents desire more program-sponsored training and access to financial resources, and this is likely transferrable to the general population of residents in training. Orthopaedic training programs should consider greater incorporation of financial education regarding personal finance, investments, and practice management and ways to build these topics into the resident education experience. Equipping residents with resources and practical knowledge on these topics will better allow residents to make informed decisions in the management of financial resources, which will serve to enhance long-term financial well-being.
